# Simultaneous bilateral patellar tendon rupture: a case report on successful repair with gracilis autograft augmentation

**DOI:** 10.1093/jscr/rjag231

**Published:** 2026-04-11

**Authors:** Luis Henrique Longo, Marcos Paulo Tercziany Vanzin, Luca Eiji Sohn Sato, Luis Antonio de Ridder Bauer, Camille Midori Okuyama, Rolmerson Robson Filho Fontes

**Affiliations:** Complexo Hospitalar do Trabalhador, 4406 Avenida República Argentina, Novo Mundo, Curitiba, PR 81050-000, Brazil; Complexo Hospitalar do Trabalhador, 4406 Avenida República Argentina, Novo Mundo, Curitiba, PR 81050-000, Brazil; Complexo Hospitalar do Trabalhador, 4406 Avenida República Argentina, Novo Mundo, Curitiba, PR 81050-000, Brazil; Complexo Hospitalar do Trabalhador, 4406 Avenida República Argentina, Novo Mundo, Curitiba, PR 81050-000, Brazil; Complexo Hospitalar do Trabalhador, 4406 Avenida República Argentina, Novo Mundo, Curitiba, PR 81050-000, Brazil; Complexo Hospitalar do Trabalhador, 4406 Avenida República Argentina, Novo Mundo, Curitiba, PR 81050-000, Brazil

**Keywords:** patellar tendon rupture, bilateral injury, extensor mechanism reconstruction, anabolic steroids, gracilis autograft

## Abstract

Simultaneous bilateral patellar tendon rupture is an exceedingly rare injury, often linked to systemic comorbidities. This report presents the case of a 31-year-old active male with a history of hypothyroidism, asthma, and chronic anabolic steroid use who sustained bilateral ruptures while running. He underwent urgent surgical repair using intratendinous sutures augmented with a double-bundle ipsilateral gracilis autograft. Postoperatively, a structured rehabilitation protocol was followed. At the 1-year follow-up, the patient achieved an excellent clinical outcome, with an IKDC score of 92.3 and a Kujala score of 95, returning to all pre-injury high-demand physical activities without limitation. This case highlights the efficacy of early surgical repair with gracilis autograft augmentation for restoring high-level knee function in patients with significant risk factors for tendon injury.

## Introduction

Patellar tendon rupture is an uncommon but debilitating injury, typically affecting active individuals under 40 [1, 2]. The simultaneous bilateral presentation is exceptionally rare, often associated with predisposing systemic conditions or pharmacological factors that compromise tendon integrity, such as chronic renal failure, diabetes, and long-term use of corticosteroids or fluoroquinolones [1, 3, 4]. Chronic use of anabolic-androgenic steroids (AASs) and pre-existing patellar tendinopathy are also significant contributors, inducing degenerative changes that render the tendon susceptible to rupture [5, 6]. This report details the successful surgical management of a young, active male with multiple risk factors who sustained a simultaneous bilateral patellar tendon rupture, using primary repair augmented with an ipsilateral gracilis autograft.

## Case report

A 31-year-old male physical education teacher and recreational weightlifter presented to the emergency department with acute bilateral knee pain and inability to stand after attempting a sprint. His history was notable for hypothyroidism, asthma, and a 3-year history of unsupervised anabolic steroid use. He also reported a 2-year history of intermittent anterior knee pain consistent with patellar tendinopathy [7].

**Figure 1:**
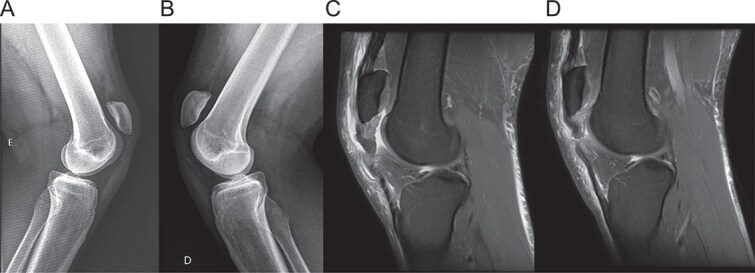
Preoperative imaging. (A, B) lateral radiographs of the right and left knee showing patella alta. (C, D) Sagittal T2-weighted MRI scans of the right and left knees, respectively, confirming complete proximal patellar tendon rupture with associated tendinosis.

Physical examination revealed significant swelling, palpable defects inferior to both patellae, and bilateral patella alta. The patient was unable to perform active knee extension. Radiographs confirmed patella alta (Fig. 1), and MRI revealed complete, full-thickness proximal ruptures of both patellar tendons with signs of chronic tendinosis (Fig. 1).

**Figure 2:**
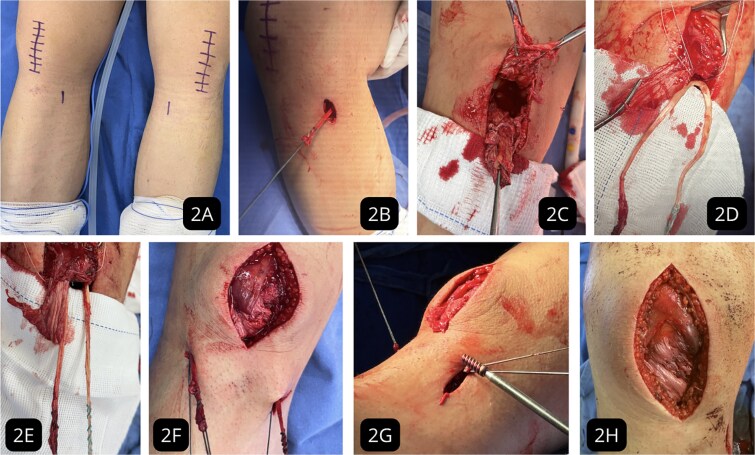
Intraoperative surgical technique. (A) Incision markings for gracilis tendon harvest and midline approach. (B) Gracilis tendon harvest. (C) Exposure of the ruptured and degenerated patellar tendon. (D) Suture anchor placement in the inferior patellar pole and passage of the gracilis autograft. (E) Double-bundle augmentation of the primary repair. (F) Creation of bone tunnels in the tibial metaphysis. (G) Distal fixation of the graft with an interference screw. (H) Final stable construct after retinacular repair.

Within 24 h of admission, the patient underwent sequential bilateral surgery. The ipsilateral gracilis tendon was harvested for augmentation. A primary repair of the degenerated tendon stumps was performed using a Krackow locking loop technique with high-strength sutures. Two 5.0 mm suture anchors were inserted into the decorticated inferior pole of the patella. The gracilis autograft was passed through the tendon substance and fixed to the anchors in a double-bundle configuration. Distal fixation was achieved with interference screws in bone tunnels created in the tibial metaphysis (Fig. 2). Graft tension was set with the knee in 30° of flexion.

**Figure 3:**
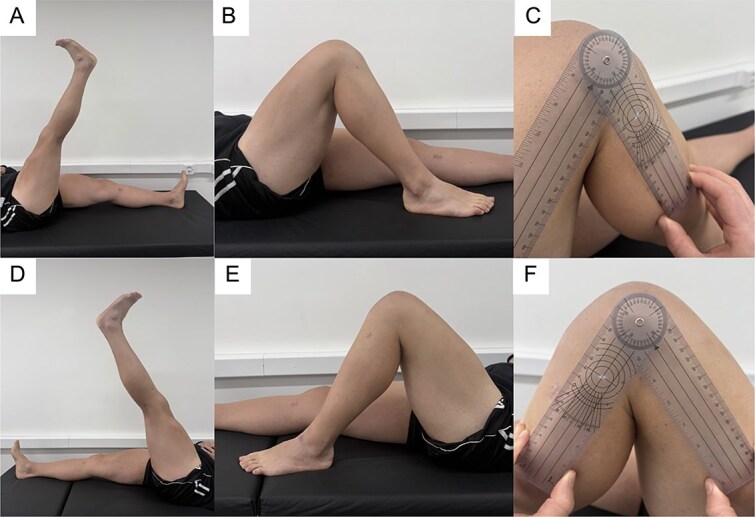
Clinical assessment at 1-year follow-up. (A, B) Active straight leg raise and knee flexion of the right knee. (C) Goniometric measurement of right knee flexion. (D, E) Active straight leg raise and knee flexion of the left knee. (F) Goniometric measurement of left knee flexion, demonstrating full and symmetric range of motion.

**Figure 4:**
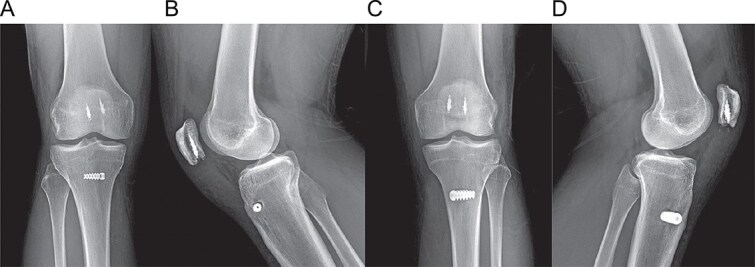
Postoperative radiographs at 1-year follow-up. (A, B) Anteroposterior and lateral views of the right knee. (C, D) Anteroposterior and lateral views of the left knee, showing stable fixation and maintained patellar height.

Postoperatively, the patient followed a structured rehabilitation protocol, beginning with partial weight-bearing in knee immobilizers and progressing to full range of motion and strengthening. At 1-year follow-up, he had returned to all pre-injury activities without limitation. His IKDC score was 92.3 and his Kujala score was 95 [8]. Examination revealed full strength and stable knees (Fig. 3). Follow-up radiographs confirmed stable fixation and maintenance of patellar height (Fig. 4).

## Discussion

This case highlights the successful outcome of early surgical repair with biological augmentation in a patient with multiple risk factors for tendon rupture, most notably chronic AAS use. The underlying patellar tendinopathy, confirmed by intraoperative findings of friable tissue, created a structurally compromised tendon. AAS use likely exacerbated this condition by creating a mismatch between rapid muscle hypertrophy and slower tendon adaptation, and potentially by directly altering the tendon matrix to reduce its tensile strength [7, 9–11].

Surgical repair is mandatory for these injuries. Augmentation is strongly recommended in cases of poor tissue quality to enhance the repair’s initial strength and allow for accelerated rehabilitation [7]. We chose an ipsilateral gracilis autograft because it provides a robust, biologically active scaffold without the risks of disease transmission or immunogenic rejection associated with allografts [12, 13]. Harvesting only the gracilis minimizes donor site morbidity compared to using the semitendinosus, which is a biomechanically stronger but functionally more significant knee flexor [14]. This approach avoids the long-term complications of synthetic materials, such as synovitis and mechanical failure [15].

The excellent functional outcome demonstrates that this technique can restore high-level function even in complex cases. Early, stable fixation combined with a structured rehabilitation protocol is critical for achieving a full return to demanding physical activities.
